# Liver cirrhosis mortality at national and provincial levels in Iran between 1990 and 2015: A meta regression analysis

**DOI:** 10.1371/journal.pone.0198449

**Published:** 2019-01-15

**Authors:** Negar Rezaei, Mohsen Asadi-Lari, Ali Sheidaei, Sara Khademi, Kimiya Gohari, Farnaz Delavari, Alireza Delavari, Elham Abdolhamidi, Maryam Chegini, Nazila Rezaei, Hamidreza Jamshidi, Pegah Bahrami Taghanaki, Milad Hasan, Moein Yoosefi, Farshad FarzadFar

**Affiliations:** 1 Department of Epidemiology, School of Public Health, Iran University of Medical Sciences, Tehran, Iran; 2 Non-Communicable Diseases Research Center, Endocrinology and Metabolism Population Sciences Institute, Tehran University of Medical Sciences, Tehran, Iran; 3 Department of Biostatistics, Faculty of Paramedical Sciences, Shahid Beheshti University of Medical Sciences, Tehran, Iran; 4 Digestive Disease Research Institute, Tehran University of Medical Sciences, Tehran, Iran; 5 School of Medicine, Dep. Of Pharmacology, Shahid Beheshti University of Medical Sciences, Tehran, Iran; 6 Faculty of Medicine, Mashhad University of Medical Sciences, Mashhad, Iran; 7 Endocrinology and Metabolism Research Center, Endocrinology and Metabolism Clinical Sciences Institute, Tehran University of Medical Sciences, Tehran, Iran; Sciensano, BELGIUM

## Abstract

**Background:**

Liver cirrhosis mortality number has increased over the last decades. We aimed to estimate the liver cirrhosis mortality rate and its trends for the first time by sex, age, geographical distribution, and cause in Iran.

**Method:**

Iranian Death Registration System, along with demographic (Complete and Summary Birth History, Maternal Age Cohort and Period methods) and statistical methods (Spatio-temporal and Gaussian process regression models) were used to address the incompleteness and misclassification and uncertainty of death registration system to estimate annual cirrhosis mortality rate. Percentages of deaths were proportionally redistributed into cirrhosis due to hepatitis B, C and alcohol use based on the data from the Global Burden of Disease (GBD) 2010 study.

**Results:**

Liver cirrhosis mortality in elder patients was 12 times higher than that in younger patients at national level in 2015. Over the 26 years, liver cirrhosis mortality in males has increased more than that in females. Plus, the percentage of change in age adjusted mortality rate at provincial levels varied between decreases of 64.53% to nearly 17% increase. Mortality rate has increased until 2002 and then decreased until 2015.The province with highest mortality rate in 2015 has nearly two times greater rate compare to the lowest. More than 60% of liver cirrhosis mortality cases at national level are caused by hepatitis B and C infection. The rate of hepatitis B mortality is four times more than that from hepatitis C.

**Conclusion:**

This study demonstrated an increasing and then decreasing pattern in cirrhosis mortality that could be due to national vaccination of hepatitis B program. However monitoring, early detection and treatment of risk factors of cirrhosis, mainly in high risk age groups and regions are essential. Cirrhosis mortality could be diminished by using new non-invasive methods of cirrhosis screening, hepatitis B vaccination, definite treatment of hepatitis C.

## Introduction

Liver cirrhosis is the pathologic term used for end stage liver disease, which is characterized with fibrosis of nodules [1]. It is reported as the 11^th^ leading cause of death globally and also is listed as one of the top ten causes of mortality in the USA [2, 3]. In Europe, 170000 deaths each year are attributed to liver cirrhosis [4]. According to the reports by Global Burden of Disease (GBD), the global cirrhosis mortality number almost doubled from 1990 to 2015. The same pattern is observed in populations with high, middle, and low socioeconomic status and in Eastern Mediterranean region countries. The mortality trend slightly increased in the mentioned countries over a period of 25 years.[3]. The mortality number reported in Afghanistan had almost tripled from 1990 till 2015. This number had approximately doubled in Turkmenistan, Armenia, Pakistan, and Iraq. On the contrary, mortality number in Turkey had decreased [3]. The main risk factors for cirrhosis are hepatitis B, hepatitis C, alcohol consumption, and nonalcoholic fatty liver disease [5, 6].

The epidemiology of liver cirrhosis mortality is reported in developed countries [7]. Unfortunately, because of the poor quality of data, this issue has not been much studied in developing countries [6]. Quantifying the problem by using statistical models helps us to develop preventive policies for managing liver cirrhosis. In addition, to the best of our knowledge there is not a comprehensive assessment of cirrhosis mortality in Iran.

To obtain an estimation of this issue for the first time, we aimed to carry out an overview of cirrhosis mortality rates from 1990 to 2015. It was also aimed to collect detailed information on risk factors such as hepatitis B and C and alcohol consumption both at national level and in thirty provinces.

## Material and methods

### Source of data

In order to calculate mortality rate, we used Iranian Death registration system (DRS) and the data obtained from the National and Sub national Burden of Diseases (NASBOD) project [8]. We addressed DRS incompleteness and misclassification by using demographic and statistical methods. In addition, age and sex and urbanization distribution of Iranian population were extracted from Iran national censuses[9]. Years of schooling (YOS) and wealth index (WI) were calculated based on the results of Iran household expenditure survey[10]. We aimed to estimate annual liver cirrhosis mortality rates, patterns, and temporal trends between 1990 and 2015 at national level and in 30 provinces in Iran.

### Definition

The International Classification of Diseases (ICD) code for underlying cause of death was used as noted in death certificate[11]. There was no criteria to evaluate the ability of physicians who filled the certificate of death. Data from ICD 10 was transformed to GBD by a physician and verified by senior physician. In this study, a percentage of deaths in subgroup B18-B18.9, I85-I85.9, K70-K70.9, K71.3-K71.51, K71.7, K72.1-K74.69, K74.9, K75.8-K76.0, K76.6, K76.7, K76.9, Z52.6, and Z94.4 were proportionally redistributed into cirrhosis due to hepatitis B, C, alcohol use, and other causes[12]. The proportions were calculated based on the data obtained from the GBD 2010 study [12, 13]. Iran national population 2015 was considered as the standard population in this process to compare Iran subnational regions with each other.

### Demographic modeling

To address incompleteness of Iranian Death Registration System in terms of child mortality, we used Complete Birth History (CBH), and Summary of Birth History (SBH). To analyze SBH, Maternal Age Cohort (MAC) and Maternal Age Period (MAP) methods were applied. Generalized Growth Balance (GGB), Synthetic Extinct Generation (SEG) and a mixture of two methods (GGB-SEG) were used for adult’s mortality incompleteness. More detailed information is presented elsewhere [14, 15].

### Statistical modeling

Briefly, spatio-temporal model was used to address misalignment in age, space and time of data. Gaussian process regression (GPR) is used to extrapolate all-cause (age and sex specific) mortality rates with a more consistent uncertainty [16–18]. The fraction of each causes of death was exerted in all-cause mortality and was extrapolated using spatio-temporal model. A simulation approach was used to estimate the uncertainty of predicted values. This mutual process is a valid statistical method that is defined elsewhere [19, 20].

### Misclassification correction

After addressing all-cause mortality rates, cause-specific rates were divided based on cause fractions extracted from the original data. More detailed information is presented elsewhere [19, 20]. For calculating the cause specific liver cirrhosis mortality, we divided the number of deaths for each cause of cirrhosis into all the same year's cirrhosis deaths in the same provinces and also in national level, and then we calculate the proportion of each of the causes to the total cirrhosis deaths. All graphs and maps were prepared using R statistical software version 3.1.2 [21]. In addition, to apply direct age standardized approach, we used “Epitools” package in R software [22].The more detail on the above mentioned method is available on S1 File.

## Results

**Fig 1:**
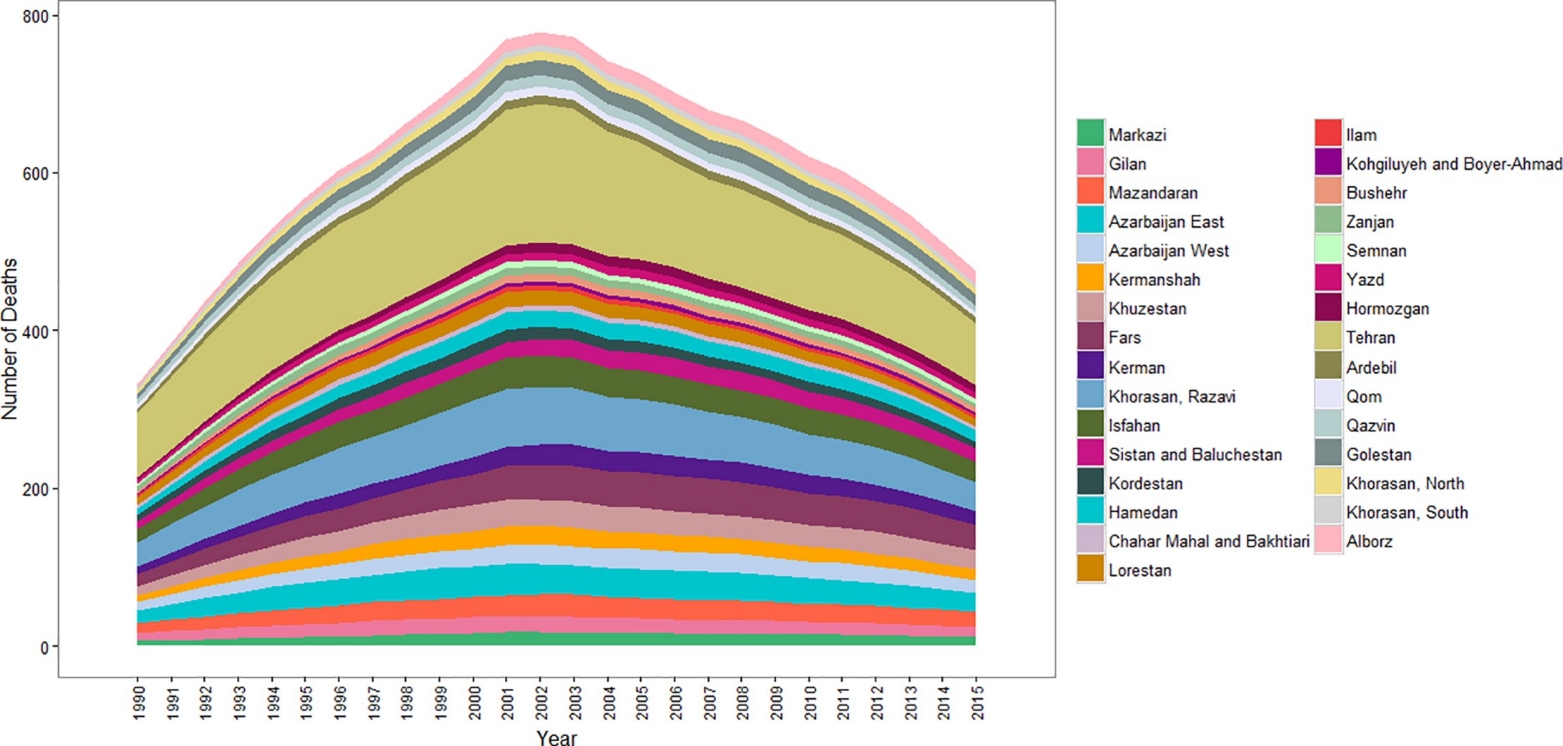
Liver cirrhosis absolute number of deaths from 1990 to 2015 by province in Iran.

Between 1990 and 2015, the absolute number of cirrhosis deaths was initially increased until 2002, and then it decreased in 2015 (Fig 1 and S1 Table).

**Fig 2:**
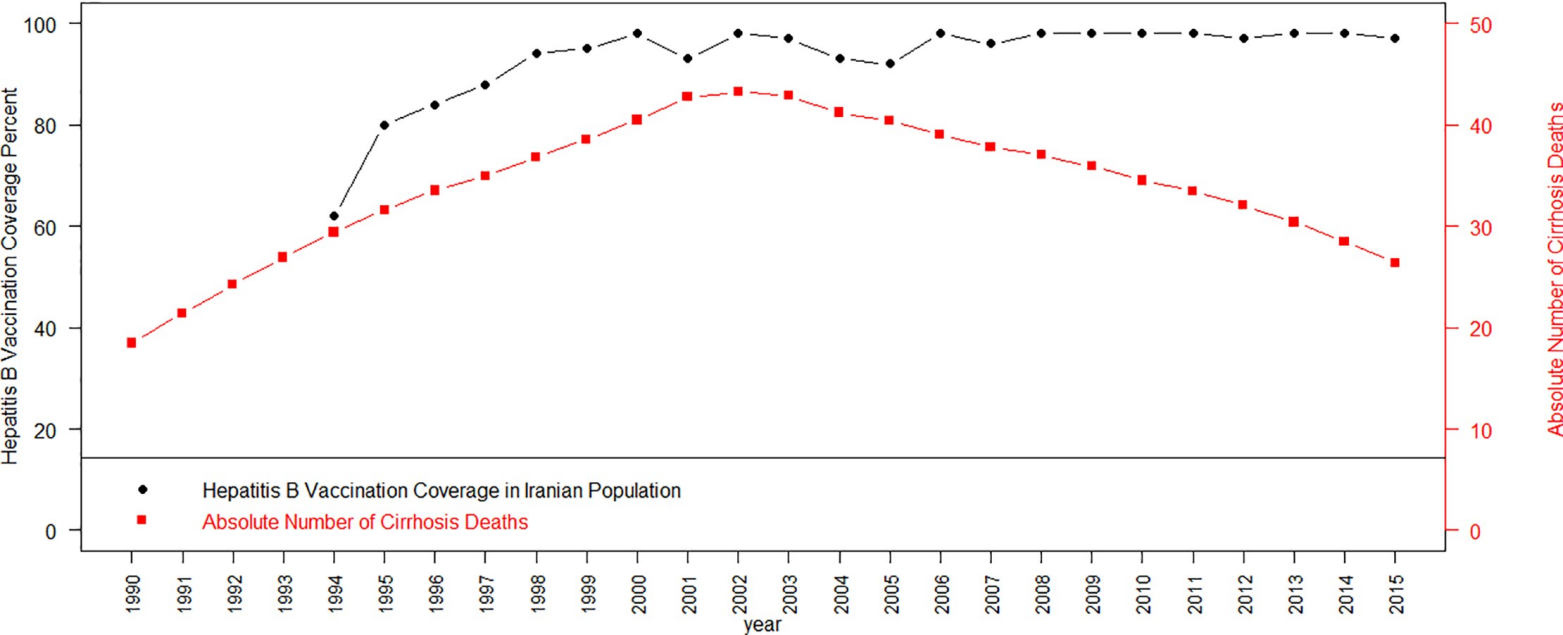
Percentage of hepatitis B vaccination coverage in Iran from 1993 to 2015 and absolute number of deaths due to cirrhosis in Iran.

Vaccination of hepatitis B was initiated in 1993 and reached the maximum level of percentage of coverage in 2000 at national level. Absolute number of cirrhosis mortality was increasing till 2002 and then decreasing at national level. (Fig 2)

There was a 39% reduction in age-standardized cirrhosis mortality rate from 0.99 (95% CI: 0.77–1.27) per 100000 in 1990 to 0.60 (95% CI: 0.48–0.75) per 100000 in 2015 at national level. (Table 1).

**Table 1:** National and provincial age standardized mortality rate in Iran (per 100,000) for both sexes and the percentage of change (Δ).

National/p provincial	1990(95% CI)	2000(95% CI)	2015(95% CI)	% Δ 1990 to 2015	ANNUALLY PERCENT CHANGE
**National**	**0.99(0.77–1.27)**	**1.50(1.21–1.87)**	**0.60(0.48–0.75)**	**-39.39**	**-1.52**
Markazi	0.90(0.73–1.11)	1.65(1.38–1.97)	0.77(0.63–0.93)	-14.44	-0.56
Gilan	0.82(0.66–1.02)	1.17(0.97–1.42)	0.5(0.41–0.62)	-39.02	-1.50
Mazandaran	0.87(0.7–1.09)	1.20(0.98–1.48)	0.59(0.47–0.75)	-32.18	-1.24
Azarbaijan East	0.90(0.73–1.11)	1.47(1.22–1.78)	0.63(0.52–0.77)	-30.00	-1.15
Azarbaijan West	0.79(0.64–0.98)	1.18(0.98–1.41)	0.5(0.42–0.61)	-36.71	-1.41
Kermanshah	0.96(0.79–1.16)	1.69(1.42–2.01)	0.7(0.58–0.84)	-27.08	-1.04
Khuzestan	0.57(0.46–0.7)	1.10(0.91–1.34)	0.5(0.41–0.61)	-12.28	-0.47
Fars	0.73(0.59–0.9)	1.32(1.09–1.59)	0.67(0.55–0.83)	-8.22	-0.32
Kerman	0.82(0.68–1)	1.22(1.03–1.45)	0.56(0.46–0.68)	-31.71	-1.22
Khorasan, Razavi	1.10(0.88–1.37)	1.83(1.50–2.24)	0.58(0.47–0.72)	-47.27	-1.82
Isfahan	0.79(0.62–1.01)	1.17(0.93–1.45)	0.51(0.4–0.64)	-35.44	-1.36
Sistan and Baluchestan	0.96(0.73–1.25)	1.20(0.94–1.53)	0.63(0.5–0.79)	-34.38	-1.32
Kordestan	0.95(0.75–1.19)	1.52(1.26–1.83)	0.55(0.46–0.66)	-42.11	-1.62
Hamedan	0.98(0.81–1.18)	1.63(1.38–1.92)	0.82(0.68–0.98)	-16.33	-0.63
Chahar Mahal and Bakhtiari	0.99(0.81–1.22)	1.24(1.03–1.49)	0.52(0.43–0.64)	-47.47	-1.83
Lorestan	1.22(0.99–1.52)	1.66(1.39–1.99)	0.55(0.45–0.66)	-54.92	-2.11
Ilam	1.01(0.82–1.26)	1.77(1.44–2.16)	0.66(0.53–0.82)	-34.65	-1.33
Kohgiluyeh and Boyer-Ahmad	0.91(0.71–1.17)	1.29(1.03–1.62)	0.5(0.4–0.63)	-45.05	-1.73
Bushehr	0.70(0.56–0.87)	1.32(1.09–1.59)	0.52(0.42–0.64)	-25.71	-0.99
Zanjan	1.30(1.03–1.66)	1.57(1.31–1.87)	0.47(0.36–0.61)	-63.85	-2.46
Semnan	1.00(0.78–1.28)	1.59(1.28–1.98)	0.87(0.69–1.1)	-13.00	-0.50
Yazd	0.93(0.75–1.15)	1.41(1.15–1.72)	0.65(0.53–0.81)	-30.11	-1.16
Hormozgan	0.53(0.42–0.67)	1.07(0.88–1.31)	0.62(0.49–0.78)	16.98	0.65
Tehran	1.72(1.23–2.39)	2.12(1.57–2.88)	0.61(0.45–0.83)	-64.53	-2.48
Ardebil	0.92(0.75–1.13)	1.25(1.05–1.50)	0.58(0.48–0.7)	-36.96	-1.42
Qom	1.02(0.77–1.34)	1.43(1.10–1.84)	0.54(0.42–0.69)	-47.06	-1.81
Qazvin	1.23(1.00–1.50)	1.74(1.46–2.08)	0.68(0.57–0.83)	-44.72	-1.72
Golestan	0.87(0.70–1.07)	1.54(1.29–1.84)	0.73(0.6–0.89)	-16.09	-0.62
Khorasan, North	1.22(0.97–1.53)	1.90(1.55–2.33)	0.77(0.62–0.95)	-36.89	-1.42
Khorasan, South	0.94(0.74–1.19)	1.60(1.29–1.98)	0.8(0.64–1)	-14.89	-0.57
Alborz	0.59(0.46–0.77)	1.11(0.88–1.40)	0.64(0.51–0.81)	8.47	0.33

**Fig 3:**
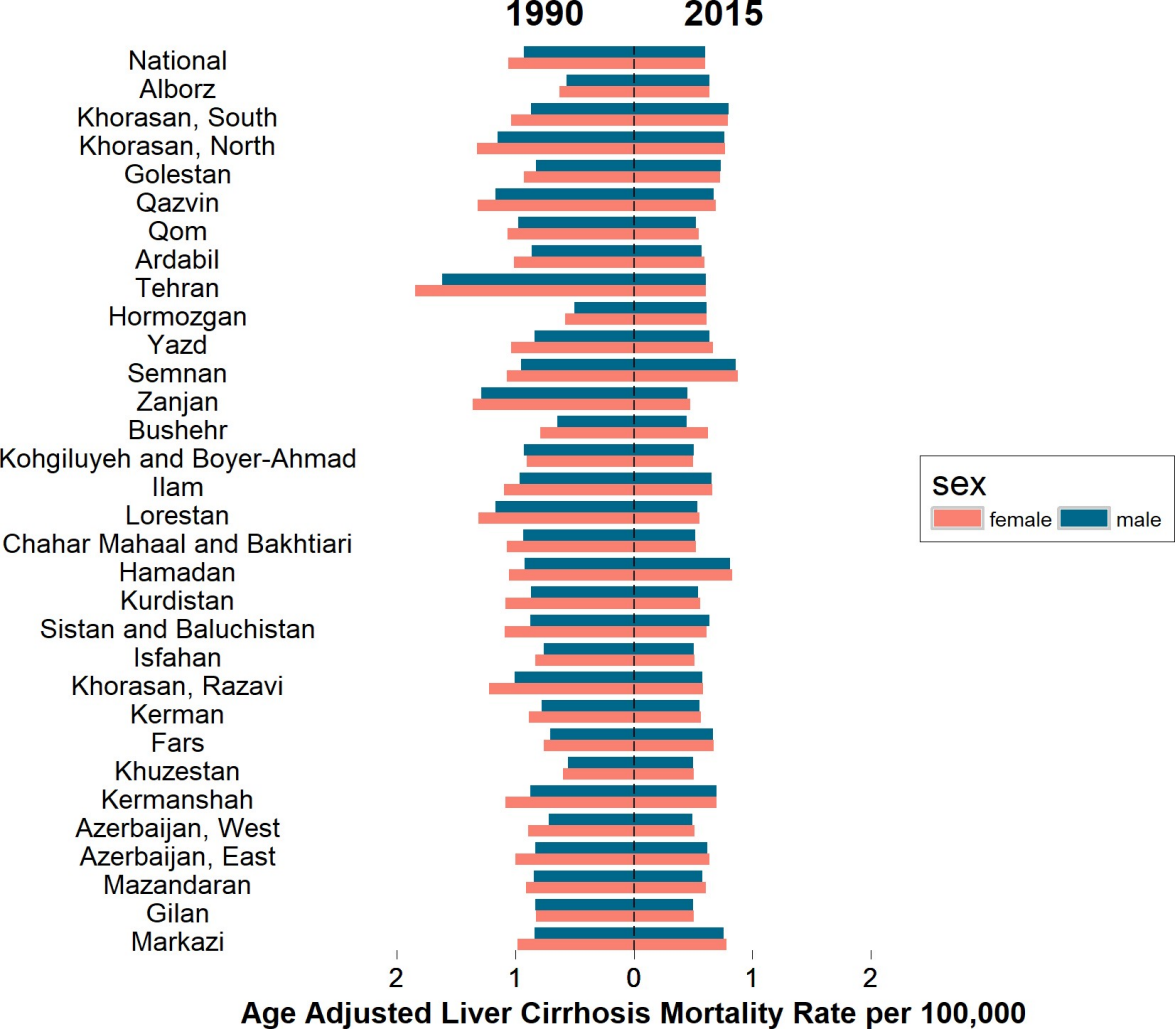
Age adjusted mortality rate in females and males by province in Iran between 1990 and 2015.

It seems that death from liver cirrhosis in male has increased faster than that in females over the 26 years. In 1990 male to female age adjusted mortality ratio was approximately 0.88 and in 2015 it increased to approximately 0.99 (Fig 3).

**Fig 4:**
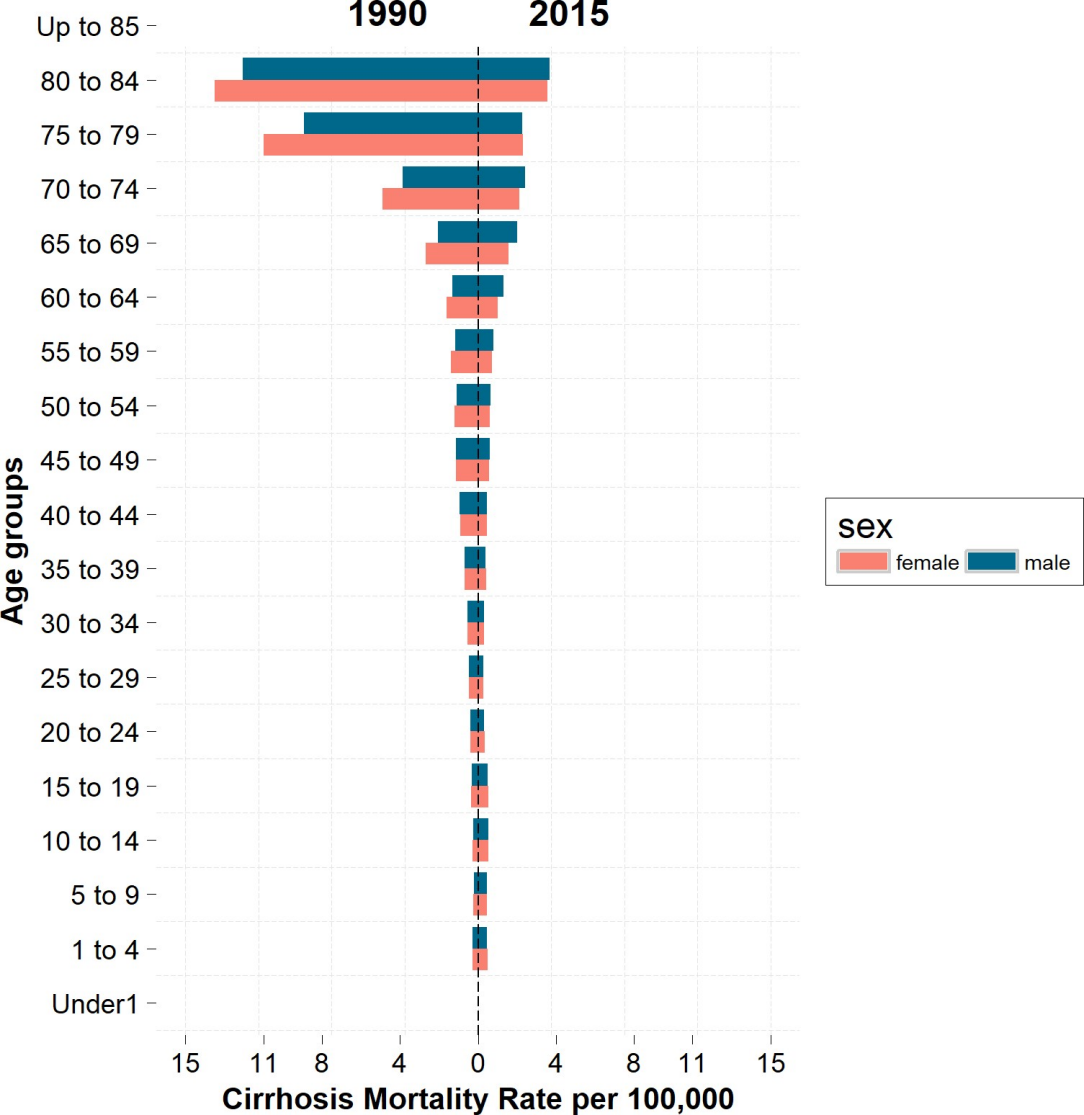
Age adjusted mortality rate in females and males by age in Iran between 1990 and 2015.

At national level, the rate of cirrhosis mortality in the age group 80 to 85 years was approximately 12 times more than that in the age group 30 to 35 in 2015. This increase was even higher in the age group 85 years and older by nearly 36 times. The pattern of increasing mortality is similar across all age groups in 1990. (Fig 4). The percent change of mortality increased by 54% in the age group under 20 years while it decreased by 46% in the age group over 20 years.

**Fig 5:**
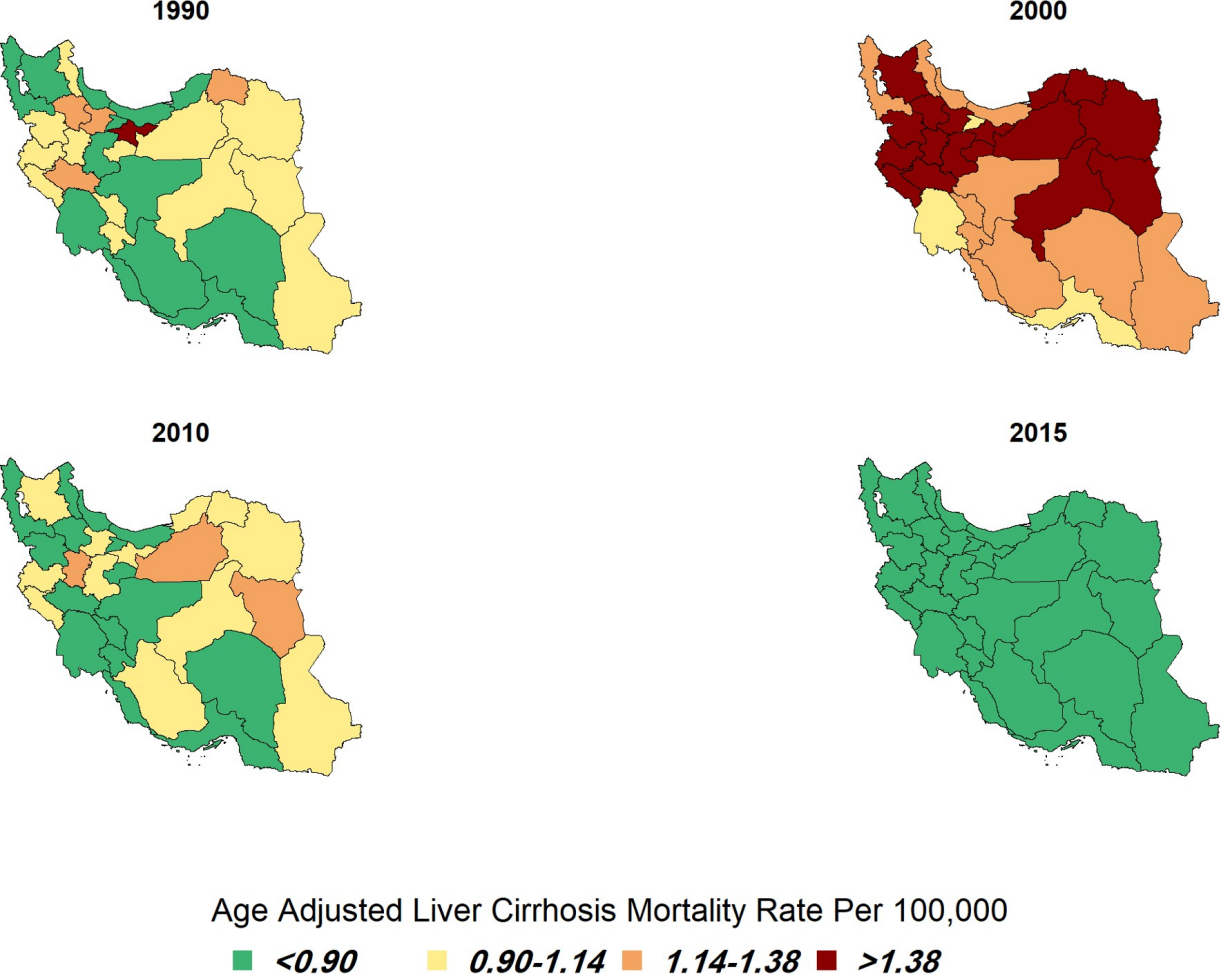
Age-adjusted liver cirrhosis mortality (per 100,000) for both sexes in 1990, 2000, 2010, and 2015 in Iran.

The percentage of change in age adjusted mortality rate at provincial levels varied between a decreases of 64.53% to nearly 17% increase, over the 26 years. Although most of provinces had decreeing rate during 26 years, two provinces showed an increasing pattern in mortality rate. (Table 1). The lowest age-standardized liver cirrhosis mortality rate in 1990 was observed in southwest of Iran with 0.53(95% CI: 0.42–0.67) per 100000. Age-standardized liver cirrhosis mortality rate increased to its highest level in the mid-2000s. The north east part of Iran had the highest age-standardized liver cirrhosis mortality rate in 2000 with 1.90(95% CI: 1.55–2.33) per 100000. Finally in 2015, all rates decreased to the least at the provincial level with 0.47(95% CI: 0.36–0.61) per 100000 (Table 1 and Fig 5).

The age adjusted cirrhosis mortality rate at provincial level was higher in females than in males in 1990 but it was closely similar in both sexes in 2015 (Fig 3).

**Fig 6:**
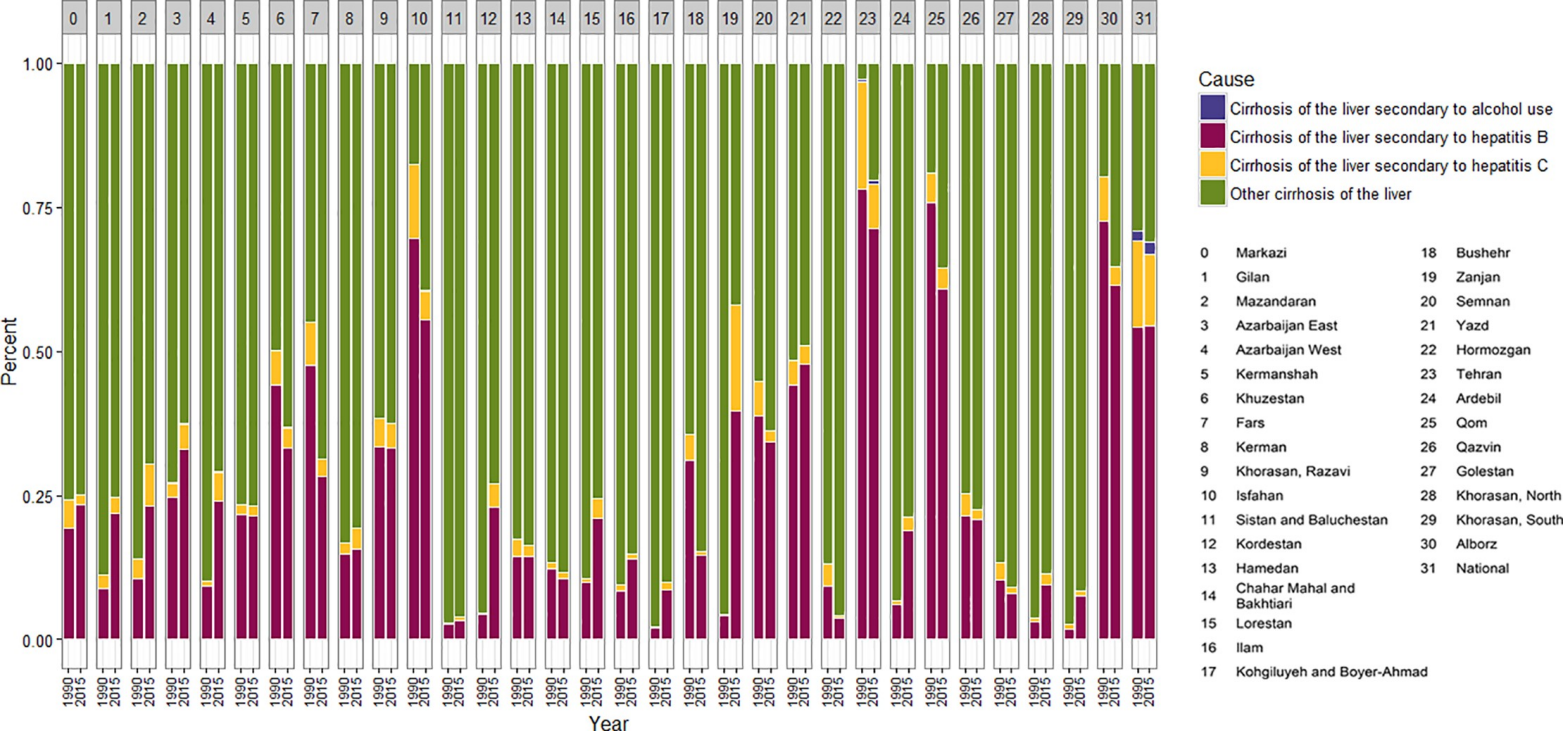
Cause specific liver cirrhosis mortality, age adjusted rate by province in Iran between 1990 and 2010.

Generally, more than 60% of liver cirrhosis mortality at national level is due to hepatitis B and C infections in 2015. However, hepatitis B and C in 1990 were responsible for approximately 70% of deaths. On the average, liver cirrhosis mortality rate due to hepatitis B was four times more than that of hepatitis C. At provincial levels, the distribution of mortality causes varied. In 2015, hepatitis B accounted for a range of 3% to 75% of liver cirrhosis mortality in different provinces (Fig 6).

**Fig 7:**
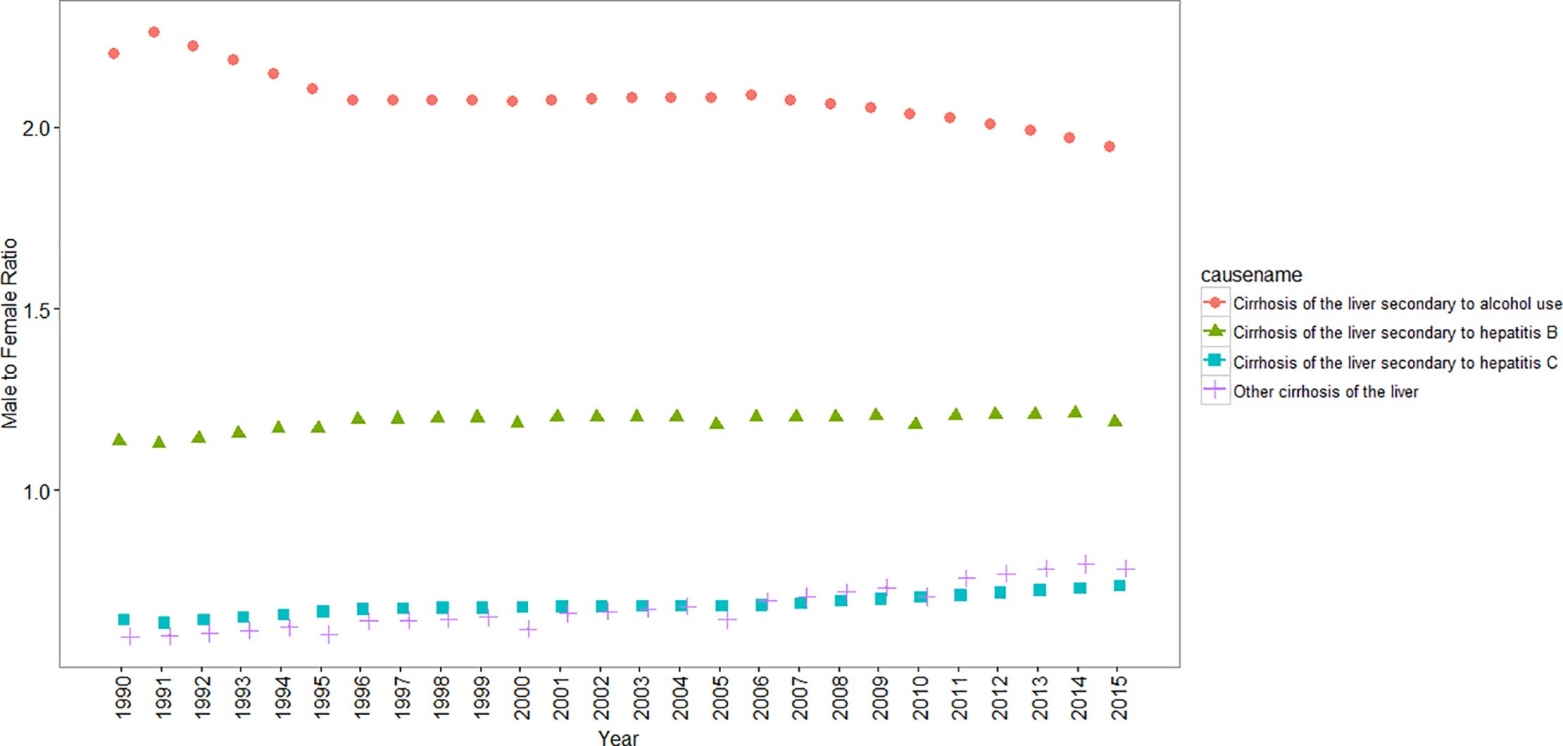
Male to Female age-adjusted liver cirrhosis mortality ratio between 1990 and 2015 by cause in Iran.

Liver cirrhosis mortality attributed to alcohol consumption was much more common in males. In 1990, the ratio of male to female age adjusted mortality attributed to alcohol consumption was 2.20; in some provinces, it even reached three. In 2015, male to female age adjusted mortality ratio decreased to less than two. Liver cirrhosis mortality due to hepatitis C was more common in females. Male to female age adjusted mortality ratio due to hepatitis C was 0.64 in 1990 which increased to 0.74 in 2015. (Fig 7)

## Discussion

This study showed that, based on Iranian data, liver cirrhosis mortality at national level increased by 12 times in older age group (80 to 85) in 2015; as compared with the younger age group (30 to 35). More than 60% of liver cirrhosis mortality at national level was due to hepatitis B and C infection. However, the distribution at subnational level varied consistent with the prevalence of risk factors. On the average, the rate of liver cirrhosis mortality due to hepatitis B was four times more than that of hepatitis C. To validate this study results, we have used the most globally up to date demographic and statistical modeling for counting incompleteness [14, 15] and cause distribution of mortality and uncertainty [13, 17, 19, 20]. The values obtained in this study are surprisingly much lower than those reported by available international records of Iran in GBD and related published articles [3, 6]. After assessing the GBD reports we did not find any specific data point of cirrhosis from Iran; probably, to predict cirrhosis mortality rate of Iran, GBD used the data from neighboring countries which had very high rates of cirrhosis mortality like Afghanistan, Armenia and Pakistan, using statistical prediction modeling [6, 23]. To the best of our knowledge, there is no published article on cirrhosis mortality rate in Iran which collected its data via population-based cohorts, but our results are consistent with the reports of liver cirrhosis registry on this issue [24]. Besides, hospitalization data on cirrhosis in Iran, which is under publication, have consistent results and proves our estimates.

The age adjusted mortality rate of cirrhosis are decreasing in countries located in East Asia (eg. China, North Korea and Taiwan), North Africa and Middle East (eg. Saudi Arabia, Iran, Iraq, Lebanon, Morocco, Egypt), and South Asia (eg. Bangladesh, India, Pakistan). This is in line with the global trend and our findings. Conversely, in Central Asia (eg. Armenia, Azerbaijan, Tajikistan, and Turkmenistan) and Eastern European countries (eg. Belarus, Estonia, Russia, and Ukraine) this trend is increasing [3, 6, 7]. Afghanistan is a neighbor country with the highest standardized cirrhosis mortality rate in the region; it was 52.53 (34.9–68.61) in 2015.

Among gastrointestinal disease, cirrhosis of liver and hepatic failure are the third main causes of mortality in Iran. Likewise, the most prevalent gastrointestinal disease diagnosed in a reference hospital in Tehran was cirrhosis of liver and hepatic failure. [25, 26]. The fact that liver cirrhosis mortality increased in older age groups is predictable and probably reflects the natural history of cirrhosis and aging process. An individual exposed to some factor related to the disease (viral hepatitis, alcohol, metabolic syndrome and other diseases) takes about 20 years to develop cirrhosis. In addition, aging increases the possibility of the fibrotic response formation[27]. So, the older groups in 2015 represent people that have been exposed to these exposures in the past and that were probably not submitted to interventions such as vaccination. In fact, this is one of the great difficulties in studying cirrhosis, based on Iranian data.

According to this study findings, the hepatitis B infection is a major cause of cirrhosis at national and subnational levels. Hence, hepatitis B control strategies must be the top concern of health policy makers. The hepatitis B vaccination program, which is a preventive health strategy, was established in 1993 as a routine child vaccination program[28]. The effectiveness of this program is obvious due to dropping rate of hepatitis B prevalence in Iran to 3% (95% CI: 2% to 3%) in 2014[23] to 2.2% (95% CI: 1.9% - 2.6%) in 2016[29]. According to our results, the cirrhosis death number had increasing pattern until 2000 and then decreased to minimum level till 2015. This pattern is compatible with maximum coverage of vaccination of hepatitis B in Iran with a lag of time (Fig 2). It seems that the vaccination program has been effective in reducing cirrhosis mortality. In China the reduction of liver cirrhosis mortality was due to hepatitis B which is in line with Iran’s pattern of mortality [6, 30]. Possible underlying risk factors contributing in chronic hepatitis incidence in Iran are reported as: unsafe surgical and dental procedures, unsafe sexual activity, intravenous (IV) drug use, blood transfusion, male gender, some particular occupations, and using shared contaminated syringes in prisons[31, 32].Hence, promoted strategies must be considered in designing future prevention policies; for example training workshops on preventing high risk behaviors. Currently, the availability of new short term treatments for hepatitis C has resulted in complete cure of many patients with chronic HCV infection [33]. Cost effective evaluation of providing these treatments for reducing cirrhosis mortality due to hepatitis C in high risk provincial level in Iran is essential. The growing epidemic of obesity in Iran may have an increasing role in liver cirrhosis due to “other causes” in the past two decades. Studies have suggested the increasingly significant impact of nonalcoholic cirrhosis on cirrhosis mortality [34, 35]. However it is difficult to be quantified based on our data and future studies are needed on this issue. It is important to reevaluate nutritional programs of community based on available studies by health policy makers to reduce the impact of diet on cirrhosis[36]. At this time noninvasive methods are available for early detection of cirrhosis [37, 38]. It is important for health authorities to utilize essential screening tools for high risk groups.

Liver cirrhosis due to alcohol consumption is an underling issue in Europe and Latin America, but in Pacific and Middle East most of the cirrhosis mortality is attributed to hepatitis B and hepatitis C infection [1, 39–41]. For example, Egypt has the highest prevalence of hepatitis C in general population and other population groups. So, the standardized mortality rate of cirrhosis is correspondingly the highest globally by 103.09(83.32–111.83) in 2015 [31, 42]. Because of religious inhibitions, alcohol consumption is not permitted in Iran, therefore alcohol use is not considered as a major cause of cirrhosis mortality.

Assessing the incompleteness, misclassification, cause distribution, and finding garbage and null codes of mortality data are the most important limitations of many mortality studies[43, 44]. As the strength of this study, we addressed this issue by utilizing internationally demographic and statistical modeling’s which were mentioned above. As another limitation of this study, cirrhosis and hepatocellular carcinoma have same major causes such as alcohol consumption, hepatitis B and C; consequently the mortality rate may be underestimated. Also, increase in deaths in those under 20 years age group could be an artifact of small numbers However we have used the statistical methods to removes overlaps as much as possible. The findings of this study are also consistent with hospitalization reports on cirrhosis at national and provincial levels which were mentioned above. As another strength of this study, we calculated uncertainty of mortality rates at national and provincial levels using appropriate statistical methods.

## Conclusion

To the best of our knowledge, this study is the first report of cirrhosis mortality trend at the national and provincial levels during a period of 26 years in Iran. It showed that it is required to control and screen cirrhosis and common risk factors in high risk age groups. This report is useful for authors, grant claimants, funding organizations, and policy makers. It could be also used for future research on burden of this disease which is important for medical education and policy making. The health care system needs national and subnational estimates of disease mortality to establish health development programs. Cirrhosis mortality could be diminished by using new non-invasive methods of cirrhosis screening, hepatitis B vaccination, curable treatment of hepatitis C, and reducing obesity via designing nutritional policies and programs at national and subnational levels particularly in high risk groups.

## Supporting information

S1 Table Absolute number of cirrhosis deaths (95% uncertainty intervals) in 1990, 2000, 2010, and 2015at national and provincial levels. (PDF)

S1 File Statistical methods. (PDF)
