# Supplementary material for: Liver cirrhosis mortality at national and provincial levels in Iran between 1990 and 2015: A meta regression analysis
Source: PLoS One. 2019 Jan 15;14(1):e0198449. doi: 10.1371/journal.pone.0198449 (PMC6333345; doi:10.1371/journal.pone.0198449)
Supplement: S1 Table — (PDF) [file pone.0198449.s001.pdf]

Absolute number of cirrhosis deaths (95% uncertainty intervals) in 1990, 2000, 2010, and 2015at national and provincial levels.				
**National/Provincial**	**1990**	**2000**	**2010**	**2015**
**Iran**	332.24(259.75-425.70)	728.40(585.84-907.42)	620.98(500.18-771.88)	475.18(379.28-595.61)
Markazi	6.33(5.13-7.80)	16.57(13.86-19.79)	14.54(12.12-17.44)	11.33(9.33-13.74)
Gilan	10.09(8.14-12.49)	20.35(16.84-24.58)	15.71(12.90-19.10)	12.84(10.43-15.78)
Mazandaran	12.51(10.03-15.60)	25.20(20.44-31.03)	23.10(18.47-28.83)	18.94(14.90-24.05)
Azarbaijan East	16.89(13.69-20.82)	38.31(31.76-46.21)	32.78(27.15-39.55)	23.94(19.70-29.13)
Azarbaijan West	10.45(8.43-12.96)	23.08(19.26-27.65)	21.03(17.56-25.16)	16.30(13.51-19.64)
Kermanshah	8.73(7.20-10.59)	22.25(18.69-26.47)	17.92(15.00-21.39)	13.95(11.57-16.82)
Khuzestan	10.84(8.81-13.34)	32.76(27.00-39.72)	28.38(23.42-34.78)	23.76(19.44-29.01)
Fars	14.93(12.06-18.48)	39.10(32.34-47.25)	39.78(32.69-48.37)	32.55(26.39-40.11)
Kerman	9.68(7.99-11.72)	21.39(18.03-25.37)	23.27(19.41-27.88)	17.97(14.76-21.85)
Khorasan, Razavi	31.08(24.95-38.70)	72.32(59.19-82.40)	51.65(42.00-63.51)	36.32(29.21-45.09)
Isfahan	17.62(13.83-22.45)	37.71(30.20-47.03)	32.99(26.40-41.19)	26.12(20.71-32.92)
Sistan and Baluchestan	9.59(7.32-12.54)	18.37(14.43-23.38)	21.09(16.78-26.47)	16.34(12.97-20.55)
Kordestan	7.10(5.65-8.92)	15.62(12.99-18.80)	12.03(10.13-14.30)	8.48(7.06-10.19)
Hamedan	8.91(7.36-10.77)	20.60(17.45-24.30)	18.80(15.86-22.28)	14.78(12.31-17.72)
ChaharMahal and Bakhtiari	4.11(3.35-5.03)	7.22(5.99-8.69)	6.18(5.11-7.47)	4.81(3.94-5.87)
Lorestan	9.83(7.93-12.17)	19.04(15.91-22.78)	13.88(11.67-16.50)	9.81(8.14-11.81)
Ilam	2.48(1.99-3.07)	6.15(5.02-7.53)	4.80(3.89-5.92)	3.82(3.07-4.73)
Kohgiluyeh and Boyer-Ahmad	2.22(1.73-2.86)	4.84(3.86-6.07)	4.47(3.58-5.57)	3.48(3.58-5.71)
Bushehr	3.24(2.60-4.03)	8.39(6.95-10.12)	7.88(6.47-9.59)	6.71(5.41-8.32)
Zanjan	6.24(4.92-7.93)	10.81(9.03-12.92)	7.25(5.76-9.11)	4.92(3.76-6.42)
Semnan	3.17(2.47-4.06)	7.02(5.62-8.74)	6.96(5.57-8.68)	5.76(4.56-7.26)
Yazd	4.53(3.66-5.61)	9.65(7.88-11.80)	8.96(7.28-11.01)	7.50(6.04-9.30)
Hormozgan	3.30(2.62-4.16)	9.88(8.10-12.05)	11.40(9.23-14.09)	10.58(8.40-13.32)
Tehran	81.63(58.58-113.55)	157.29(116.06-213.36)	112.12(83.14-151.13)	78.18(57.62-105.87)
Ardebil	5.49(4.45-6.76)	10.38(8.67-12.41)	10.11(8.48-12.05)	7.36(6.12-8.87)
Qom	5.04(3.83-6.63)	10.74(8.32-13.82)	8.42(6.56-10.79)	6.55(5.09-8.42)
Qazvin	6.59(5.38-8.07)	13.60(11.41-16.21)	11.78(9.85-14.08)	8.55(7.08-10.31)
Golestan	7.00(5.68-8.60)	17.73(14.81-21.21)	17.69(14.53-21.36)	13.89(11.37-16.97)
Khorasan, North	4.80(3.83-6.02)	10.55(8.59-12.93)	8.97(7.31-11.00)	7.05(5.68-8.74)
Khorasan, South	3.68(2.91-4.65)	7.36(5.95-9.09)	7.18(5.77-8.90)	5.40(4.30-6.77)
Alborz	4.11(3.18-5.29)	14.11(11.18-17.77)	18.92(15.12-23.69)	17.16(13.59-21.63)
