# Supplementary material for: Liver cirrhosis mortality at national and provincial levels in Iran between 1990 and 2015: A meta regression analysis
Source: PLoS One. 2019 Jan 15;14(1):e0198449. doi: 10.1371/journal.pone.0198449 (PMC6333345; doi:10.1371/journal.pone.0198449)
Supplement: S1 File — (PDF) [file pone.0198449.s002.pdf]

Study design

Target population is divided into 19 age groups consisting of children under one year old, one to four years old, and then 5-year age groups and 85+ group. All processes were done for males and females separately.

Causes of death were registered based on tenth International Classification of Disease (ICD10) codes but we convert the ICD 10 into Global Burden of Disease (GBD) study. Exploration of GBD codes shows us that 170 of Iran's ICD 10 codes are eligible to be considered GBD cause of death for Iranian population.

Our study included all deaths related to Iranian residents who were living in Iran. In addition, records related to cases of mutilation (buried limbs), cases of abortion or stillbirth, foreigners who were not resident of Iran and duplicates, all were eliminated. Finally, 3,645,608 individual records of death formed the primary data set.

Data source

1- Provincial DRS data from 1995 to 2001, which were collected by Deputy for Research and Technology (Data set 1995-2001)
2- Provincial DRS data from 2001 to 2004, which were collected by Deputy for Public Health (Data set 2001-2004)
3- Provincial and district DRS data from 2006 to 2010, which were collected by Deputy of Public Health (Data set 2006-2010)
4- Behesht_zahra cemetery data from 1995 to 2010 (Tehran data)
5- Baghe_rezvan cemetery data from 2007 to 2010 (Isfahan data)

Calculating the cause specific death rate by sex and age groups in each province needs appropriate population data. We have extracted it from the national censuses of 2011, 2006 and 1996. For other years, the following growth model was used to estimate population data [1]:

$$P = P_0 e^{rt}$$

At first, for each consecutive censuses, growth rate (r) was calculated by considering first census as initial population (P_0) and second one as final population (P). Then population was obtained by changing time variable (t) for years between two censuses. This process was conducted in each sex and age groups and all provinces.

Censuses and Household Expenditure and Income Survey (1985-2013) provide useful information about distribution of socio-economic and cultural variables across the country. Among them, we created and applied covariates such as wealth index, years of schooling and urbanization in our statistical modeling. These are variables, which affect cause of death and were available to include in our study[2].

Data issues:

Although DRS became an ongoing process in the recent years, it is not yet appropriate to be used in order to scientific objectives. Following issues are those that should be solved before conducting any analysis:

Inconsistency in DRS administration: There are several institutions, which have administered DRS in the last 20 years. Deputy of Research and Technology in MOHME have established DRS and administered data for seven years since 1995. After that, Deputy of Public Health in MOHME took responsibility for administering DRS since 2001. On the other hand, Tehran and Isfahan cemeteries that are administering their deaths data independently. Coding systems, diseases categories, age groups, and coverage of DRS varies from one institution to another one. In order to aggregate data sets, all variables, which are essential in cause of death study, were selected. Then other information in each data set was used to improve validity of these variables. Finally, variables were recorded to same codebook and main data set was formed by combining uniform data sets.

Duplicates: There are several kinds of duplicates in Iran DRS Data sets. The first happens when an individual death is registered several times. It usually happens when a person who is resident of city A travels to city B to receive medical treatments and dies and will be recorded as two separate death events in both cities. On the other hand, it is usual to bury limbs with its owner characteristics so mutilation is another reason for registering a case more than once. The other type of duplicates is duplication among data sets. For instance, considerable percent of Tehran and Isfahan cemeteries records are in MOHME data sets too. Sometimes it is possible to recognize duplicates and remove them from data set especially for individual records. For second type of duplicates, we had to select more reliable data set.

Misalignment: Administrative divisions of Iran's provinces and districts have been reformed several times in last 25 years. It means, some adjacent districts have become independent from one province and formed a new province or joint to an existing province. Therefore, we chose administrative division in 2011 (31 provinces) as the reference and restructured data sets of other years to have all 31 provinces for all years.

Misclassification: DRS in Iran encounters high rate of misclassification in different types. Misclassification of cause of death is the most important one. Age-sex restriction (the mismatch between registered age, sex, and cause), garbage, and ill-defined codes are other kinds of misclassification. Geographical misclassification (provinces) is fairly related to misalignment Using prior information is a common and an advised approach to solve misclassification problem [3].

Missing values: Dealing with missing values is critical to the future analysis. We should explore the nature of missing values including pattern, type, and cause of them

Incompleteness: Incompleteness is the main issue, not only in DRS but also in all registration systems. Even the best DRS cannot capture all deaths so incompleteness is an important criteria in validity assessment of DRSs. Mohamadi et al are conducting a parallel study on level and trend of mortality in Iran from 1990 to 2014 and we used their outputs to adjust our results especially applying rate of incompleteness [4].

Lost space-time data points: Despite of cited issues, there are space or time points without any valid registered data. For instance, national DRS data set is not available in 2005 .Also there are several provinces have not registered death records in some years. Furthermore, the present registration system did not collect data for all periods of study (from 1990 to 1994), so we have to estimate results for these lost space-time data points.

Because of issues in DRS data, studying cause of death is a critical and time-consuming process. Firstly, a wide range of methods was applied for preparing data in order to future analysis. Secondly, we used several statistical models to estimate mortality rates by age-sex-province groups for all causes of death. Variety of target groups and causes of death made model selection and evaluation of results more complicated. Knowledge about all steps of these processes could be useful in similar experiments so we bring them in detail. Graphical illustration of these steps is also available in figure 1.

Data preparation:

Step 1: Key variables of this study including age, sex, cause of death, residency area, and nationality were extracted from all data sets. Sex and nationality variables were recoded to unique values. Unusual values were registered for age in all data sets because of insensitive data entry process. That is why we recoded ages more than 115 into missing values. Age 115 was chosen because above this age is unlikely age in Iranian population [5].

We formed new provinces based on provinces in administrative division 2011 for each data set:

1- In data set 1995-2001which was based on catchment areas of each Medical University was assigned to existing provinces in 2011.
2- In data set 2001-2004 provinces were reformed same as data set 1995-2001 and there was no case of mismatch.
3- In data set 2006-2010, we used district variable to reshape provinces based on administrative division in 2011. If district was missing, province of residency and Medical University that death happened in its catchments area were used respectively to fill in the district gap.

4- In Tehran data set, we assumed that all records were related to Tehran or adjacent cities so we considered them as deaths of Tehran province residents.
5- In Isfahan data set, we assumed that all records were related to Tehran or adjacent cities so we considered them as deaths of Isfahan province residents.

Finally, there was no missing value in province variable.

An expert physician in ICD10 advised us in exploring cause of death. Seventeen chapters of ICD10 were eligible to be considered in studying cause of death including chapter 1-19 except chapter 7 (diseases of eye and adnexa) and chapter 18 (Symptoms, signs and abnormal clinical and laboratory findings, not elsewhere classified) [6]. Wrong codes like "it will be specified later" or "YYY" and garbage codes in chapter 18 of ICD10 were recoded into missing value. In Tehran, causes of death were registered according to diagnosis not ICD10. A team of physicians assigned ICD10 codes to diagnosis and several possible codes in unclear cases.

In many cases, garbage codes, or aggregation of several codes were registered as underling cause. Possible ICD10 codes were considered in these situations. We provided a frame of age and sex restriction for all causes. It had been assumed that registration of wrong cause of death is more likely than wrong age or sex. Based on this frame, we recoded impossible causes of death into missing values. We did an exception in data set 2006-2010 to deal with sex restriction by checking first name if it was necessary.

Lastly, we reached three following kinds of cause of death: 1.Specified underling cause with ICD10 code, 2.missing value when we have no information about underling cause and 3. Several possible ICD10 codes according to incomplete information.

Step 2: In step 1, all data sets were combined and formed the main data set. In the present step, we aimed to impute all missing values. We used a multiple imputation approach using Amelia package [7] in R software for age and sex variables and a multinomial imputation using STATA 11 software [8] for cause of death. In addition, since majority of records were related to Iranian resident (more than 99%). we assigned missing value in nationality variable to be Iranian.

Amelia performs multiple imputations a general-purpose approach to deal with missing values. Multiple imputations can reduce bias and increase efficiency compared with other methods [9]. Amelia also uses a bootstrap and EM algorithm to impute missing values from a data set [7]. This approach produces multiple output data sets for analysis. We introduced main data set to Amelia as a time series data. Year was considered as time variable and province as cross section variable. Imputation was done in the presence of age, sex, and dummy variables of ICD10 chapters as covariates. The value of 115 was considered as maximum level of age variable and number of imputation was set on five.

After each imputation, we checked age and sex restrictions. Imputed values of age or sex were recorded into missing value if there was any restriction. Amelia imputed missing values of rechecked data set again. This process had been done until no missing value and age-sex restriction exist in data set. At the end of this stage, we combined 5 age and sex imputed versions of primary data set.

We applied a multinomial imputation approach including two stages to impute cause of death variable. At first stage, missing values in cause of death variable were assigned into ICD-chapters using the following multinomial logistic model:

$$\Pr(x_i = k|z_i) = \begin{cases} \dfrac{1}{1+\sum_{l=2}^{k}\exp(z_i'\beta_l)}, if\ k = 1 \\ \dfrac{\exp(z_i'\beta_k)}{1+\sum_{l=2}^{k}\exp(z_i'\beta_l)}, if\ k > 1 \end{cases}$$

Where z_i is the vector of predictors for observation i and β_l is regression coefficient for outcome $l = 2, \dots, K$. Here we used age, sex and year as covariates. At the second stage, the related causes were imputed in each ICD chapter. To avoid imputing unacceptable cause, a process of checking age-sex restriction was done after second stage. Causes of death that had been detected in this process were recoded into missing value. We continued this approach to the extent that there was at least a missing value in cause of death.

Step 3: As it was mentioned, we had three situations for cause of death variable after step 1: first, cause of death matched to specific underling cause and ICD10 code; second, missing values in cause of death were imputed in step 2 and finally, uncertainty in cause of death according to garbage codes. In this step, we aimed to redistribute deaths of the latter situation to other specified cause of death.

We first calculated number of death by age, sex, province, cause, and year. Then we redistributed deaths of garbage codes on deaths of probable cause by using proportionality approach in related age, sex, and cause combination in each year. There were situations that proportion of death could not be calculated in some age and sex combinations so we eliminated age effect on death distribution.

Step 4: Making comparable results needs standard classification codes for causes of death. Although ICD10 is the most popular classification disease approach, GBD codes was preferable because of comparing results with GBD study. An expert physician familiar to definition of diseases and causes of death explored definition of all codes in GBD and ICD10. As the result, a map was created to identify relation between codes in two classification systems. Finally, comparing GBD and ICD10 showed three kinds of relation between codes:

1- ICD10 classified cause of death in more detail than GBD. In this case, we had several codes in ICD10, which are corresponding to one code in GBD. We called them many to one (MTO) codes. Dealing with MTO codes easily conducted by aggregating deaths related to many codes in ICD10 into one code in GBD.
2- ICD10 and GBD had same definition for a cause of death and allocated one code for that. We called these codes one to one (OTO) codes. We only recoded these codes.
3- GBD classified cause of death in more detail than ICD10. The main issue in recoding causes of death into GBD codes was related to this situation. Thus, we had an ICD10 code (OTM codes) that should be assigned to several GBD codes proportionally.

A proportional redistribution approach was considered to deal with OTM codes. This approach needed the prior distribution of GBD cause of death in each age, sex, and year. This information was available for some years in GBD study. For other years, we predicted number of death using the following Poisson regression model with logarithm link function. In this model, years of schooling (YOS), wealth index (WI) and urbanization ratio were considered as covariates. In addition, logarithm of population was added to model as offset to adjust results.

$$\begin{aligned}\mathrm{Log}(number\ of\ death\ for\ cause\ i) \\ = \alpha + \log(population) + \beta_1 sex + \beta_2 age + \beta_3 year + \beta_4 YOS + \beta_5 WI \\ + \beta_6 urbanizratio\end{aligned}$$

Step 5: Mohamadi et al. have conducted parallel study on the same data sets in order to predict levels and trends of child and adult mortality rates in the Islamic Republic of Iran from 1990 to 2013. They applied a spatial temporal model and Gaussian process regression to predict rates of death for all provinces of Iran [4]. We used their results to deal with incompleteness of DRS. According to their results; we just had all-causes mortality rates in 31 provinces for males and females. Therefore, we had to assume that incompleteness of all causes is equal and the rates of death by the same scale parameter for all causes of death are changed.

All cause specific mortality rates were rescaled by above-mentioned approach except rates of death in Alborz. This province is separated recently from Tehran in 2010. Therefore, many cases of death related to this province were registered in Tehran. Exploring trend of incompleteness in Alborz confirms this issue. Unfortunately, high rate of misclassification in majority time period of study caused sparse or empty cells in Alborz data set. In this situation scale up could not solve this problem. We used cause distribution

of Tehran and Alborz in each age and sex groups and applied predicted rate of death for Alborz to identify death distribution in this province. Necessary assumption in this approach was equality of death distribution according to age, sex, and cause in both provinces. We hope that this assumption was satisfied because of adjacency of two provinces and similarity of death covariates.

Step 6: After above five steps we had a unique data set. Death rate by age, sex, province and cause was determined based on locations and years which DRS captured data points. For estimating lost location-time data points, three modeling approaches were applied consisting of a random intercept mixed effect model and two spline model with 0.6 and 0.9 smoothing parameters.

Random effect: For all causes in all combination of age and sex levels, we modeled logarithm of death rates per 100000 by the following mixed effect model:

$$\log(rate) = \alpha + b_i + \beta_1 year + \beta_2 YOS_{ij} + \beta_3 WI_{ij} + \beta_4 urbanizratio_{ij} + \beta_5 log\big(Population_{ij}\big) + \varepsilon_{ij}$$

$$b_i \sim N(0, \sigma_b^2) \text{ , } \varepsilon_{ij} \sim N(0, \sigma_\varepsilon^2)$$

Where ε_{ij} is the residual for province i and year j and b_i is the random effect related to province i. By using the random effects part in this model, we were able to explore effect of each province separately.

Spline model: Spline is a nonparametric approach to smooth complicated functions. We assumed values 0.6 and 0.9 for smoothing parameter in each combination of age, sex to predict trend of each cause in provincial and national level.

Step 7: We used three different modeling approaches for causes of death according to the strength and nature of available data. To select the best-fitted model, we used five criteria including statistical and epidemiological inferences. Statistical criteria explored goodness of fit and detected outliers. On the other hand, the trends were compared between age groups and years to find unusual patterns for all causes and provinces. These criteria are summarized below:

1. Normalized root mean square deviation (NRMSD): This criteria measured the goodness of fit and is equal to root mean square deviation divided by range of observation:

$$NRMSD = \frac{\sqrt{\frac{\sum_{t=1}^{n}(\hat{y}_t - y)^2}{n}}}{y_{max} - y_{min}}$$

2. Outlier according to observed data: We prepared a confidence interval for observed data in each age-sex group for all causes and provinces. It is defined as the three standard deviations before and after the mean of observation. For each model, we expected that no predicted value should be out of this range. Therefore, we count predicted values that did not satisfy this criteria for each cause.

3. Outlier according to predicted values: Sometimes several predicted values come outside the range of other predicted values. To find these outliers, we defined an indicator, which took value one if a predicted value was outside the range of three standard deviations from means of other predicted values. Our criteria were calculated by summing indicator values by each cause.

4. Two-sequence age groups proportion: To compare trends between age groups we assume that mean of mortality rate for specific cause and sex in an age group should not be five times more or less than adjacent age groups. Number of age groups for each cause that did not satisfy this assumption was considered as the criteria.

5. Slope of trends: This criterion was defined by this assumption that the mean of each five-year mortality rates should not be three times less or more than mean of mortality rates in adjacent five years. Again, an indicator was introduced to count unsatisfactory situation for each cause. This process was done for the first and the last five years period.

Finally, the best model for each cause was selected based on combination of all these criteria.

Step 8: After modeling, it is necessary to have an equivalent relation between levels of study. For instance, total number of death at sub-national level should be equal to number of deaths at national level. In addition, there should not be any differences between total number of death according to cause specifics and all-causes. It seems to have more appropriate results in all-cause and national level comparing with cause specific and sub-national level respectively because of contributing more data points in modeling these levels.

A two-step approach was used to equalize the scale of different levels. At first, each cause-specific mortality rates were scaled up or down to have equal summation to all-cause mortality rate. In the second stage, rescaling was done for sub-national cause-specific and sub-national all-cause mortality rates.

Step 9: All estimations are reported with a value of uncertainty. We calculated uncertainty of our estimates by a simulation approach. After determining model for all causes, steps 5 and 6 were done 1000 times. Instead of point estimation, random normal values with mean of point estimation and standard deviation equal to standard error of predicted mortality rates in step 5 were applied. Finally, we had 1000 point estimations therefore the 2.5th and 97.5th percentiles of these values constructed lower and upper bound of uncertainty interval respectively.

References:

1. Keiding N. Extinction and exponential growth in random environments. Theoretical Population Biology. 1975;8(1):49-63.
2. Lozano R, Naghavi M, Foreman K, Lim S, Shibuya K, Aboyans V, et al. Global and regional mortality from 235 causes of death for 20 age groups in 1990 and 2010: a systematic analysis for the Global Burden of Disease Study 2010. The Lancet. 2013;380(9859):2095-128.
3. Khosravi A, Rao C, Naghavi M, Taylor R, Jafari N, Lopez AD. Impact of misclassification on measures of cardiovascular disease mortality in the Islamic Republic of Iran: a cross-sectional study. Bulletin of the World Health Organization. 2008;86(9):688-96.
4. Mohammadi Y, Parsaeian M, Farzadfar F, Kasaeian A, Mehdipour P, Sheidaei A, et al. Levels and trends of child and adult mortality rates in the Islamic Republic of Iran, 1990–2013; protocol of the NASBOD study. Archives of Iranian medicine. 2014;17(3):176-81.
5. Vakilian K, Khadijeh Mirzaii N. Reproductive health in iran: international conference on population and development goals. Oman medical journal. 2011;26(2):143.
6. Organization WH. International Classification of Diseases (ICD). 2013. Available from: apps who int/classifications/apps/icd/icd10online [last accessed 13 Apr 2011]. 2011.
7. Honaker J, King G, Blackwell M. Amelia II: A program for missing data. Journal of Statistical Software. 2011;45(7):1-47.
8. White IR, Royston P, Wood AM. Multiple imputation using chained equations: Issues and guidance for practice. Statistics in medicine. 2011;30(4):377-99.
9. Honaker J, King G. What to do about missing values in time-series cross-section data. American Journal of Political Science. 2010;54(2):561-81.
